# In-Line Fiber Optic Interferometric Sensors in Single-Mode Fibers

**DOI:** 10.3390/s120810430

**Published:** 2012-08-02

**Authors:** Tao Zhu, Di Wu, Min Liu, De-Wen Duan

**Affiliations:** Key Laboratory of Optoelectronic Technology & Systems (Ministry of Education), Chongqing University, Chongqing 400044, China; E-Mails: wudi@cqu.edu.cn (D.W.); liumin@cqu.edu.cn (M.L.); ddw99@126.com (D.-W.D.)

**Keywords:** in-line fiber-optic sensor, Fabry-Perot interferometer, core-cladding-mode interferometer, Mach-Zehnder Interferometer, Michelson interferometer

## Abstract

In-line fiber optic interferometers have attracted intensive attention for their potential sensing applications in refractive index, temperature, pressure and strain measurement, *etc.* Typical in-line fiber-optic interferometers are of two types: Fabry-Perot interferometers and core-cladding-mode interferometers. It's known that the in-line fiber optic interferometers based on single-mode fibers can exhibit compact structures, easy fabrication and low cost. In this paper, we review two kinds of typical in-line fiber optic interferometers formed in single-mode fibers fabricated with different post-processing techniques. Also, some recently reported specific technologies for fabricating such fiber optic interferometers are presented.

## 1. Introduction

Optical fiber sensors have been widely used in sensing applications of various physical, chemical, and even biological measurements, owing to their compact, small size, fast responses, high resolution, high sensitivity, good stability, good repeatability, and resistance to electromagnetic interference. In the past few decades, fiber grating optical technologies, including fiber Bragg gratings (FBG) and long-period fiber gratings (LPFG), have attracted a lot of attention. Grating-based (FBG, LPFG) sensors, which have the advantages of high sensitivity and high multiplexing capability, have been used to sense strain, pressure, temperature, refractive index (RI), polarization, *etc.* [1–13]. Meanwhile, fiber optic interferometer sensors also have been widely deployed owing to their special characteristics. So far, four typical types of interferometers, including Fabry-Perot, Mach-Zehnder, Michelson, and Sagnac, have been demonstrated in optical fibers. A fiber optic interferometer operates on the interference effect between two beams that propagate through different optical paths of a single fiber or two different fibers. It can be seen that the beam splitting and combining components are required in any configuration of the fiber optic interferometers.

For Fabry-Perot interferometers (FPIs), two parallel separated mirrors are required to partially reflect the lead-in optical signals. The typical FPI can be categorized into two types: one is the extrinsic FPI in which the cavity is external to the fiber and the other is intrinsic FPI in which the cavity is contained within the fiber. In general, the cavity of the extrinsic FPI can be formed by the air [14–17] or a polymer [18–23]. Due to the small thermal expansion coefficient of air, air-cavity based extrinsic FPIs could be used as temperature-insensitive sensors for pressure or refractive index (RI) or strain sensing. On the other hand, since polymers can have special characteristics, polymer-cavity based extrinsic FPIs can be designed for special requirements, such as high temperature sensitivity, molecule detection, *etc.* Overall, although the fabrication of the extrinsic FPI is relatively simple and low-cost, the extrinsic FPI sensors have low coupling efficiency, require careful alignment, and have packaging problems. On the other hand, the sensing element of the intrinsic FPI is a short section of fiber sandwiched between two reflecting components. To form the local cavity of the intrinsic FPI, several techniques have been introduced, such as internal film coating [24,25], refractive-index mismatch between two fibers in the splicing joint [26–28], fiber Bragg grating (FBG) [29], laser irradiated points [30–32], chemical etching [33–35], *etc.* Since the light signal of an intrinsic FPI propagates in the fiber all the time, a higher intensity optical signal will be obtained, which is better for signal demodulation. However, for these intrinsic FPIs, there still exist the disadvantages of requiring expensive equipment for the cavity fabrication or needing special fibers or using dangerous chemical reagents. In particular, the LPFGs are well known to be sensitive to the ambient variations, and simultaneous measurement of RI and temperature could be realized by cascading the LPFG and the intrinsic or extrinsic FPI [36,37]. Moreover, the definition of the intrinsic and extrinsic FPI becomes vague owing to the advent of specialty fibers and fiber devices. For example, a FPI could be formed by sandwiching a section of hollow core fiber (HCF) whose core is air between two fibers, in which the cavity is not only the fiber but also the air [38–45]. In this case, due to the hollow core of the HCF, various substances could be fed into the cavity of the FPI, such as by injecting liquids or gases with different RIs into the cavity to measure RIs with high sensitivity [45], or by filling a polymer into the cavity to realize a high sensitivity temperature measurement [43]. Recently, photonic crystal fibers (PCF) have attracted extensive interest all over the World because of their complex pattern of microscopic air-holes in the transverse plane that runs along the fiber. Since the holey structure gives PCFs unique guiding mechanisms and modal properties that are impossible with conventional optical fibers, there is much research on FPIs based on PCFs [46–51].

For the Mach-Zehnder interferometer (MZI), Michelson interferometer (MI), and Sagnac interferometer (SI), the incident light is split into two arms by a fiber splitter and then recombined by a fiber combiner. Early MZIs and MIs had two independent arms, which functioned as the sensing arm and the reference arm, respectively [52–54]. The main differences between MZI and MI are that the MI requires only one coupler to be the splitter/combiner and mirrors to reflect the two beams at the end of each arm. Particularly, the two arms of the SI are, in fact, an optical fiber loop. The two beams are propagating along the loop in counter directions with different polarization states [55,56]. These fiber optic interferometers with two arms have their own limitations, such as complicated structure, big size, high susceptibility to environmental fluctuations, *etc.* However, an in-line fiber optic core-cladding-mode interferometer (CCMI) has been proposed recently to replace the separated arm interferometers. The typical in-line fiber optic CCMI sensors have been demonstrated in both Mach-Zehnder and Michelson types. The CCMI operates on the interference between the core and the cladding modes, which also requires the splitter and the combiner to realize the coupling and re-coupling between the core mode and the cladding modes. The reference arm and the sensing arm of CCMI are the same optical fiber, but have different optical path owing to the modal dispersion. Thus, the CCMIs are more compact and very effective. So far, several fabrication techniques have been proposed, including long-period fiber gratings (LPFGs) [57–62], optical fiber tapers [63–68], fiber peanut-shape structure [69], misaligned spliced joint [70–73], core diameter mismatch [74–80], laser irradiated points [81–84], partially collapsing the air-holes of photonic crystal fiber (PCF) [71,85–92], *etc.*

Recently, Lee *et al.* reviewed and categorized fiber optic interferometric sensors according to their operating principles, fabrication methods, and application fields [93]. In this paper, we focus on two typical in-line fiber optic interferometers, FPI and CCMI, formed only by single mode fibers (SMFs). These interferometers have two optical paths in one physical line and one of the optical paths should be arranged to be easily affected by external perturbations. Thus, the in-line structure offers several advantages such as compactness, simplicity, easy alignment, high coupling efficiency, and high stability. In particular, since the in-line fiber optic interferometers are formed by normal SMFs, the sensor fabrication will be very low-cost. Also, some specific examples of recently reported in-line fiber optic sensor technologies based on SMFs are presented in detail to show their advantages and the great potential sensing applications.

## 2. In-line FPI Formed in SMF

In recent years, fiber in-line FPIs have received much attention for their wide range of applications. A review of early development of fiber-optic FPIs can be found in [94]. Fabricating an in-line fiber optic FPI requires the formation of two parallel separated mirrors to partially reflect the lead-in optical signals into different optical paths. To form the mirrors in SMF, numerous techniques have been introduced, such as coating the end of the fiber [22], offset structures [16,95], forming a micro-notch by use of femtosecond lasers [30–32], using chemical etching [34], splicing technology [96], *etc.* Since the two beams reflected by the mirrors have an optical path difference (OPD), the relative phase difference of the two beams could be described by:

(1) $$\Phi_{\text{FPI}}=\frac{4\pi nL}{\lambda }$$

where *λ* is the input wavelength, *n* is the refractive index (RI) of FPI cavity, and *L* is the length of the FPI cavity. When a perturbation is applied to FPI, the phase difference Φ*_FPI_* between the two beams will be influenced because the cavity length increases. The change of Φ*_FPI_* contributes to the interference shifts, which means that the FPI can be used as temperature or RI sensing.

**Figure 1.**
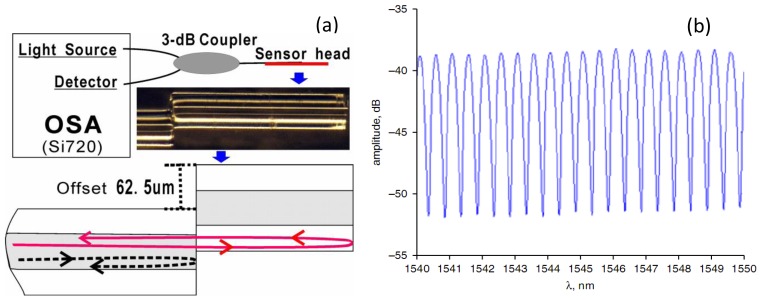
The FPI based on large lateral offset: (**a**) schematic, inset picture is the microscope photograph; (**b**) reflection spectrum.

**Figure 2.**
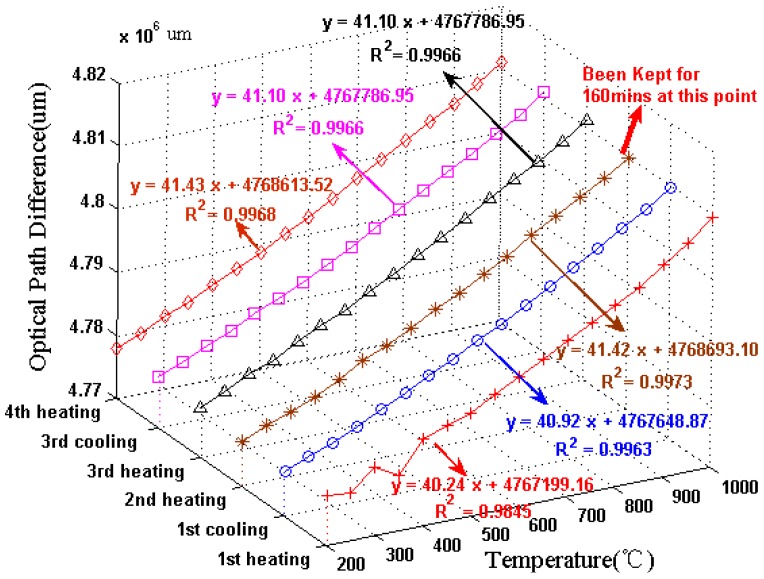
Measured reflection spectra of the FPI at different temperatures.

For temperature measurement, the in-line FPI is a popular sensor owing to its high sensitivity and good stability at high temperature. Zhu *et al.* spliced a section of special PCF to SMF to form a FPI, which could be heated up to 1,200 °C [47]. Wei *et al.* made a fiber in-line FPI with a femtosecond laser for high temperature measurement (up to 1,100 °C) [31]. Also, Choi *et al.* fabricated a FPI by fusion-splicing a short piece of holey optical fiber (HOF) between SMF and a piece of multimode fiber (MMF) to measure the temperature up to 500 °C [44]. However, these FPIs need a femtosecond laser or special optical fibers. Recently, Duan *et al.* proposed a low-cost FPI, as shown in Figure 1(a) [95]. The FPI is formed by simply splicing two sections of SMF together with large intentional lateral offset (∼62.5 μm). The formation of the FPI needs only two steps and inexpensive SMF, making it extremely low-cost. As shown in Figure 1(a), the first FPI mirror is formed due to the Fresnel reflection of part of SMF core uncovered by the next section of SMF, while the second FPI mirror is formed due to the Fresnel reflection at the next section of SMF cladding end. Figure 1(b) shows the reflective spectrum of such a FPI with a cavity geometry length *L* of ∼1.65 mm. The typical interference fringe has a visibility around 15 dB, which is sufficient for the sensing application. To characterize the high temperature response, the FPI was put into a temperature furnace whose temperature error is ±1 °C for temperature measurement. Figure 2 shows the test results of the FPI with a cavity geometry length *L* of 1.65 mm. It can be seen that in the first heating cycle, the FPI shows a poor high temperature response of the OPD; while in the later heating and cooling cycles, the linearity become better and repeatable. Thus, pre-annealing this kind of FPI up to 1,000 °C can greatly improve its linearity and the repeatability of the high temperature response. We can also see that the OPD temperature sensitivity of the FPI is ∼41 nm/°C, which is comparable with that of FPIs based on special PCFs [47].

**Figure 3.**
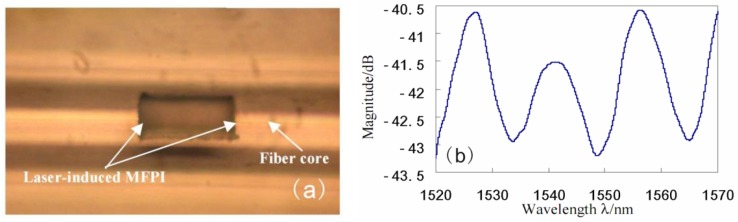
The MFPI with 80 μm cavity length based on the SMF: (**a**) microscope photograph of the cavity; (**b**) reflection spectrum.

Particularly, the cavity of in-line FPI could be formed by a femtosecond laser [30–32]. Thanks to the small laser spot of the femtosecond laser, the length of the cavity could be controlled accurately and can reach accuracy of micron order. As shown in Figure 3(a), Rao *et al.* have fabricated a micro cavity of in-line FPI (MFPI) in SMF by using a near-infrared femtosecond laser [31]. The length of the cavity is only 80 μm; the reflective spectrum of the MFPI is shown in Figure 3(b). The temperature and strain characteristics of the MFPI sensors are also studied.

**Figure 4.**
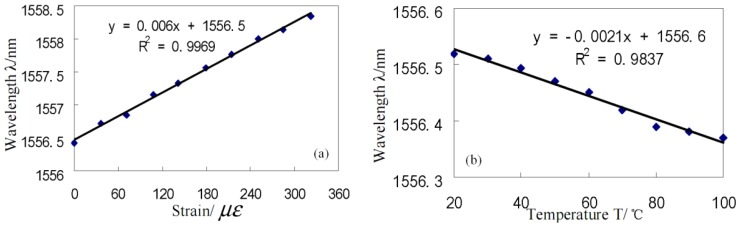
The characteristics of MFPI: (**a**) wavelength-temperature sensitivity; (**b**) wavelength-strain sensitivity.

Figure 4(a) shows that the wavelength-strain sensitivity of the MFPI sensor is ∼0.006 nm/με. In addition, it can be obtained that the phase-strain sensitivity of the MFPI is ∼2.51 × 10^−3^ rad/με, which is about five times larger than that of an in-line FPI sensor of ∼0.49 × 10^−3^ rad/με reported previously [40]. On the other hand, Figure 4(b) shows that the wavelength-temperature sensitivity of the MFPI sensor is ∼−0.0021nm/°C. Correspondingly, the phase-temperature sensitivity of the MFPI sensor is ∼−0.87 × 10^−3^ rad/°C. Hence, the temperature sensitivity of the MFPI sensor is about 11 times smaller than that of the in-line SMF etalon sensor of ∼0.01 rad/°C [39]. It should be noted that it is negative in that the two end-faces of the MFPI cavity would expand towards the cavity centre with the increment of temperature, leading to such a decrease in cavity length.

**Figure 5.**
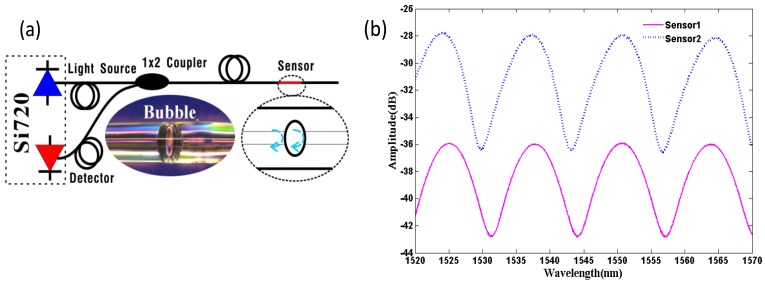
The air-bubble-based FPI sensors with cavity length of 91 μm: (**a**) schematic, inset picture is the microscope photograph; (**b**) reflection spectrum.

Although the method of using a femtosecond laser can form a micro in-line FPI with micron size, it also requires expensive equipment for the cavity fabrication. However, Villatoro *et al.*, Li *et al.* and Deng *et al.* proposed a new micro in-line FPI with micron size whose cavity is an air bubble formed by splicing PCF and SMF together [49–51]. It was found that this kind of FPIs could endure high temperatures and is relatively insensitive to external temperatures, whereas it has relative high strain sensitivity. In addition, Duan *et al.* fabricated a FPI by simply splicing two sections of SMFs together to form the air bubble at the splicing point [96]. Figure 5(a) shows a typical microscope photograph of such an air-bubble-based FPI sensor (middle bottom) formed by Duan *et al.*

**Figure 6.**
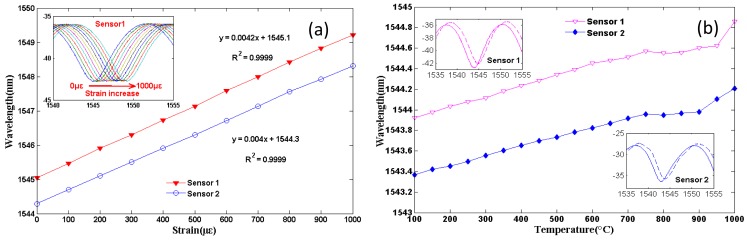
The sensing applications of the air-bubble-based FPI with 91 μm air bubble: (**a**) the strain sensitivity, inset is the shifts of one of the interference dips as the strain increases; (**b**) the temperature sensitivity, inset is the shifts of the interference dip at 100 °C (solid curve) and 1,000 °C (dashed curve).

The air bubble was realized by adjusting the splicing parameters of the commercial arc splicer (Fitel S176) to certain values. Figure 5(b) shows the reflective spectra of such two air-bubble-based FPIs with cavity length of approximately 91 μm, and the high-quality interference spectra with a fringe visibility of ∼8 dB were observed. Both the influences of strain and temperature on the reflective interference signal of the two air-bubble-based FPI sensors were experimentally studied. Figure 6(a) shows the strain responses of the two sensors under a constant temperature (∼15 °C) at the wavelength of ∼1,544 nm, inset figure is the wavelength shift of sensor 1 *versus* strain increases. The temperature response of the two sensors is shown in Figure 6(b), and the inset figure is the reflective spectra of sensor 1 and sensor 2 at 100 °C and 1,000 °C, respectively. As shown in Figure 4, air-bubble-based FPI sensors have higher strain sensitivity of ∼4.2 pm/με for sensor 1 and ∼4.0 pm/με for sensor 2, which is almost 150% higher than that of the results reported by references [49–51]. However, the temperature sensitivity of the air-bubble-based FPI sensors is only 0.848 pm/°C, which is much less than the temperature sensitivity of FBG (10 pm/°C) [3]. It can be noted that the strain sensitivity and temperature sensitivity of air-bubble-based FPI depends on the cavity length *L* and the material thermal expansion, respectively. Therefore, such an air-bubble-based FPI offers the advantage of high strain sensitivity with small temperature influence due to the larger bubble diameter (91 μm) and low thermal-expansion coefficient of pure silica (0.5 × 10^−6^/°C) [69], which makes it attractive for strain sensing applications.

Certainly, the in-line FPI could also be used for measuring the RI of liquid or gas. Up to now, many methods have been reported to realize the RI sensing based on in-line FPIs, for instance, splicing the SMF, HOF and MMF together [44], splicing the SMF and PCF together [45], using a silver layer and a vapor-sensitive polymer layer to form the two mirrors of FPI [23], *etc.* However, these RI sensors have complicated structures and low sensitivity. Therefore, Wei *et al.* described a compact FPI based on fabricating an open micro-notch cavity in a SMF by the use of femtosecond lasers [31]. Meanwhile, Duan *et al.* proposed an FPI formed by fusion splicing a short section of SMF between two sections of SMF with a large lateral offset [16]. Since the cavity of the FPIs proposed by Wei *et al.* and Duan *et al.* is open, such kind of FPIs with simple structure has higher RI sensitivity (1,163 nm/RIU fabricated by Wei, and 1,540 nm/RIU fabricated by Duan). In addition, the open-cavity structure is also beneficial to the gas RI measurement because the gas could enter the cavity easily.

## 3. In-Line CCMI Formed in SMF

Compact in-line fiber optic CCMIs are attractive for chemical, physical, and biological sensing applications. The CCMI requires a mechanism to realize the coupling and re-coupling between the modes of the fiber core and fiber cladding. The core mode is guided by the core–cladding interface of the fiber and the cladding mode is guided by the cladding-ambient interface. Due to the phase difference between the core and cladding modes, the CCMI could be used to measure many environmental parameters. The CCMI includes two types of Mach-Zehnder interferometer (MZI) and Michelson interferometer (MI).

### 3.1. In-Line MZI

In the MZI, there are a splitter to couple part energy of the core mode into the cladding modes and a combiner to recombine the cladding modes into the core. The relative phase difference of the core and cladding modes could be described as:

(2) $$\Phi_{\text{MZI}}^{m}=\frac{2\pi \Delta n_{\text{eff}}^{m}\;L}{\lambda }$$

where $\Delta n_{\text{eff}}^{m}$ is the effective RI difference between the core mode and the *m*th cladding mode, *L* is the interaction length, and *λ* is the input wavelength.

Since the core mode is well shielded by the thick cladding and the cladding modes are directly exposed to the environment, the RI of the environment can vary significantly the effective propagation constant of the cladding modes. MZIs can thus be used as RI sensors by tracking the wavelength shift of the interference fringe. In references [88,89], the RI sensors based on air-hole collapsing of the PCF have been reported. Although these PCF-based MZI sensors have several advantages including the ability to operate at high temperatures and high RI sensitivity owing to the air-holes structure, the expensive PCF will limit its mass production in industry. Thus, the techniques forming the RI sensor in SMFs is attractive because of the low cost of SMFs. To realize that, one way is to use a pair of LPFGs as the splitters/combiners to fabricate a MZI in the SMF [57–59]. The LPFG-based RI sensors have a large measuring range and high sensitivity, but they require precise (and often expensive) photolithographic alignment equipment and amplitude masks. Another way is to taper a fiber at two points along the fiber [63,66,68]. Tian *et al.* firstly proposed the MZI-based RI sensor by concatenating two SMF fiber tapers separated by a middle section [66]. Later, Lu *et al.* achieve simultaneous measurement of temperature and RI by using the same two-fiber-tapers structure [68].

**Figure 7.**
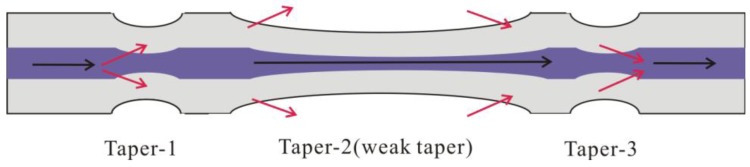
Schematic diagram of the in-line MZI based on three tapers.

**Figure 8.**
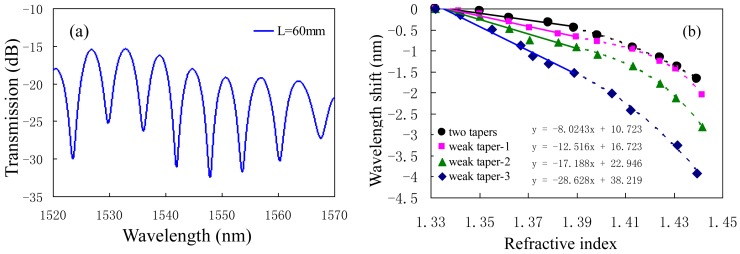
The three-tapers-based MZIs: (**a**) the transmission spectra with interaction length *L* of ∼60 mm; (**b**) the RI sensitivities with different weak tapers.

In order to improve the sensitivity to RI, Wu *et al.* introduced a MZI based on three cascaded SMF tapers [63]. As shown in Figure 7, the taper-1 and the taper-3 were used as the splitters and combiners to form a MZI, and the middle weak taper was used to increase the evanescent field of the cladding mode excited by taper-1 in the external medium. The increment is small, but sufficient for the enhancement of the RI sensitivity. Figure 8(a) shows the transmission spectrum of the three-tapers-based MZI with interaction length *L* of ∼60 mm. The detail parameters of the tapers are: the diameter and the length of both taper-1 and taper-3 were ∼20 μm and ∼6.1 mm; the diameter and the length of taper-2 (weak taper) were ∼39 μm and ∼40.8 mm, respectively. To characterize the effects of the middle weak taper on RI sensitivity, they have also fabricated other two three-tapers-based MZIs (called weak taper-1, weak taper-2), and a two-tapers-based MZI without the weak taper (called two tapers); the named weak taper-3 MZI is shown in Figure 8(a). They almost have the same parameters for the side tapers and separation lengths, however, the weak tapers in the middle are different. Weak taper-3 has the longest length and the thinnest diameter, while the weak taper-1 is converse. Experimental results show that the RI sensitivities of the four sensors were ∼80 nm/RIU (two tapers), ∼125 nm/RIU (weak taper-1), ∼172 nm/RIU (weak taper-2), and 286 nm/RIU (weak taper-3), respectively, as shown in Figure 8(b). The Equation (2) shows that the RI sensitivity of MZI could be strengthened by increasing the interferometer length *L*, however, it is not good for a compact sensor. Thus, the weak taper will play an important role in improving the RI sensitivity of the three-tapers-based MZI. The sensitivity ∼286 nm/RIU of the three-tapers-based MZI with 60 mm interaction length could be comparable with that of the LPFG pair sensor with 62 mm interaction length (259 nm/RIU) [59].

**Figure 9.**
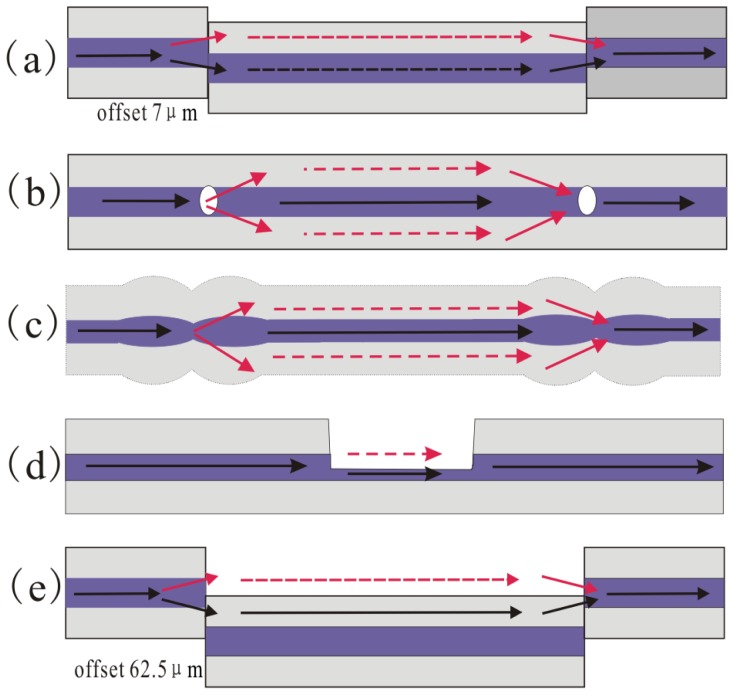
Configuration of various types of in-line MZIs; the methods of using (**a**) core-offset structure; (**b**) air-hole formed by femtosecond laser; (**c**) peanut-shape structure; (**d**) open air cavity formed by femtosecond laser; (**e**) open air cavity formed by large lateral offset splicing.

**Figure 10.**
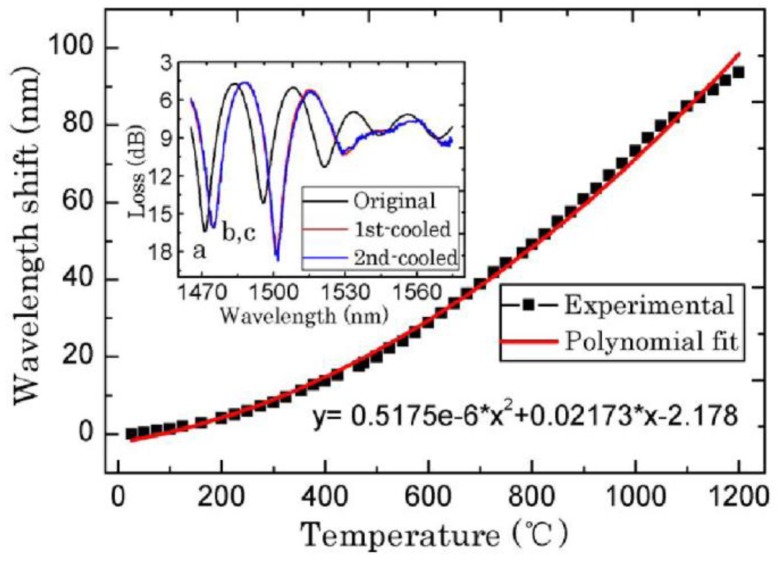
The temperature sensitivity of micro-cavities based MZI as shown in Figure 9(b). The inset is the transmission spectra (**a**) before heating at 25 °C, (**b**) and (**c**) the first time and second time of cooling down to 25 °C, respectively.

As previously stated, the in-line MZI requires the splitter and combiner to split the input optical signal into two different optical paths (the solid core and the cladding) and subsequently recombine them together. The other typical techniques for fabricating the splitter/combiner in SMF include misaligned spliced joint [70–72], peanut-shape structure [69] and laser irradiations [81–84]. As shown in Figure 9(a), the in-line MZI could be achieved by splicing two sections of SMF with 7 μm offset. The relative offset direction between the two misaligned spliced joint will affect the interferometer performance greatly. Since it is difficult to fabricate two identical offset structures, practical applications of the MZI based on 7μm core offset are limited by the low extinction ratio [70]. In reference [84], Jiang *et al.* proposed an in-line MZI based on concatenating two micro-cavities separated by 20 mm, as shown in Figure 9(b). A femtosecond laser was used to fabricate a micro-hole on the center of a fiber end. Then a micro-air-cavity was formed by splicing the micro-hole fiber end with a normal fiber end. Note that the diameter of the micro-cavity is slightly smaller than the fiber core diameter (the diameter and the depth of the hole are ∼7 μm and ∼2.5 μm, respectively), the hole with small depth will excite low-order cladding modes and make the insertion loss lower. Since the low-order cladding modes are insensitive to external RIs, the RI sensitivity of the MZI reported in reference [84] is low, while the temperature sensitivity is very high (∼109 pm/°C) in the range of 500–1,200 °C, as shown in Figure 10.

**Figure 11.**
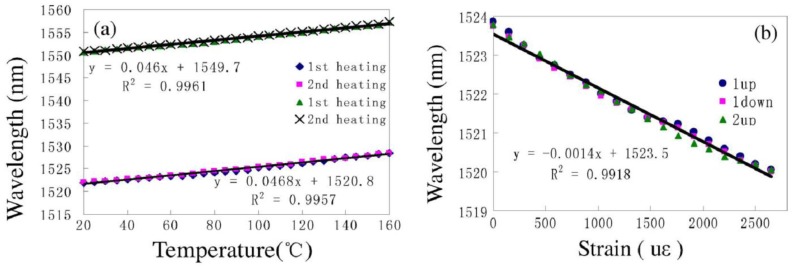
The characteristics of peanut-shape fiber structure based MZI as shown in Figure 9(c): (**a**) the temperature sensitivity; (**b**) the strain sensitivity.

In particular, reference [69] demonstrated that a novel peanut-shape fiber structure can excite high-order cladding modes and recouple the cladding modes to the core mode. The fabrication of the peanut-shape fiber structure only needs the commercial fusion splicing machine and two simple steps. Step 1 is using large arc power to make two ellipsoidal fiber microlenses; Step 2 is splicing the two ellipsoidal fiber microlenses together with normal arc power. By cascading two peanut-shape structures, a simple in-line MZI was realized, as shown in Figure 9(c). As can be seen from Figure 11, the MZI with interferometer length of ∼22 mm based on two peanut-shape fiber structures has a linear temperature sensitivity of ∼46.8 pm/°C (R^2^ = 0.9957) and a linear strain sensitivity of ∼1.4 pm/με (R^2^ = 0.9918, maximum strain is 3,670 με) when the interferometer length is ∼22 mm. Undoubtedly, it has the advantages of good mechanical strength, simplicity, and low-cost fabrication process.

**Figure 12.**
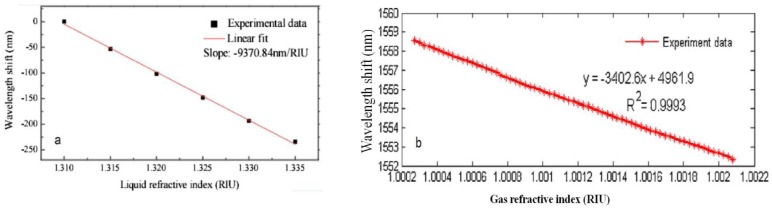
The RI sensitivity of the open cavity in-line MZI fabricated by different methods: (**a**) using femtosecond laser, as shown in Figure 9(d); (**b**) splicing with a large intentional lateral offset, as shown in Figure 9(e).

However, the above discussed in-line MZIs are based on multimode interference, the spectra are somewhat inhomogeneous since there are more than two modes involved in an inhomogeneous interference pattern. Exceptionally, the LPFG pair uses only one cladding mode in most cases. Although there is indeed one dominantly excited cladding mode to interfere with the core mode, the other weak cladding modes will still affect the sensing performance because different modes have different sensitivity to the external variations. The method to avoid the problem of multimode interference is to use the micro-cavity as one arm of the MZI, as shown in Figure 9(d,e) [72,82,83]. Figure 9(d) shows an open cavity in-line MZI based on a micro-cavity formed by using femtosecond laser to remove part of the fiber core and cladding. Meanwhile, Figure 9(e) shows another open cavity in-line MZI, in which a short section of SMF was spliced into two sections of SMFs with a large intentional lateral offset (∼62.5 μm). Such kinds of open cavity in-line MZIs have two different optical path: one is the air micro-cavity, and the other is the optical fiber (Figure 9(d) is the fiber core, Figure 9(e) is the fiber cladding, respectively). Due to the large index difference between the air cavity and the optical fiber (>0.1), a very short interferometer length can offer a large OPD, which allows a dramatic reduction of the size of MZI. Figure 12 shows that the open cavity in-line MZIs have a high liquid RI sensitivity of ∼9,370 nm/RIU in the range of 1.31∼1.335 [83], and even a high air RI sensitivity of ∼3,402 nm/RIU in the range of 1.0002–1.0022 [72].

In conclusion, these in-line MZIs introduced in Figure 9 have their own advantages and disadvantages. The small offset based MZI could be fabricated easily, while it is difficult to splice two identical offset structures to control the polarization of incident light, and practical applications of the MZI based on small offset are limited by the low extinction ratio. The two micro-cavities based MZI could be used as a high temperature sensor with high sensitivity, but requires precise (and often expensive) photolithographic alignment equipment. Due to the large waist diameter, the peanut-shape fiber structure has a good mechanical strength. Meanwhile, the fabrication of the peanut-shape fiber structure only needs the commercial fusion splicing machine to splice twice. However, the temperature or strain sensitivity of the peanut-shape fiber structure based MZI should be further increased to be applied in industry. It should be noticed that the three types of MZIs discussed above have the relative large size with millimeters and even centimeters scale. The length of the interferometers could be reduced to micron scale by using better process, but the free-spectrum range (FSR) will become very large which is against the measurement precision. On the contrary, the open cavity based MZIs can solve the problem due to the large index difference between the air cavity and the optical fiber (>0.1). A very short interferometer length with micron size can offer a large OPD, which allows a dramatic reduction of the size of MZI. The open cavity based MZIs have very high RI sensitivity because the liquid or gas can leave or enter the open cavity to be the sensing arm. However, it is necessary for the open cavity based MZIs to enhance the mechanical strength and reduce the insert loss because of the open cavity formed by removing part of the fiber or splicing with large lateral offset.

### 3.2. In-Line MI

In the in-line MIs, the interference principles are quite similar to that of in-line MZIs. The main difference between MI and MZI is that the MI only needs one fiber structure to use as the splitter and combiner, which makes it more compact and handier than MZI in practical use and installation. Since the optical signal propagates along the interference arms twice, the relative phase difference between core mode and cladding modes could be described as:

(3) $$\Phi_{\text{MZI}}^{m}=\frac{4\pi \Delta n_{\text{eff}}^{m}\;L}{\lambda }$$

where $\Delta n_{\text{eff}}^{m}$ is the effective RI difference between the core mode and the *m*th cladding mode, *L* is the interaction length, and *λ* is the input wavelength.

**Figure 13.**
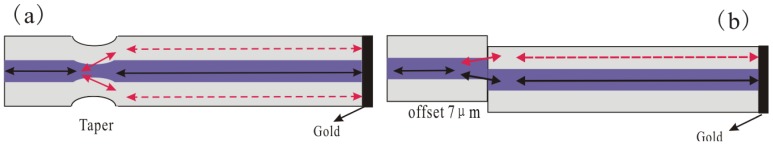
Schematic of two in-line MIs based on: (**a**) the fiber taper; (**b**) the core-offset structure, respectively.

**Figure 14.**
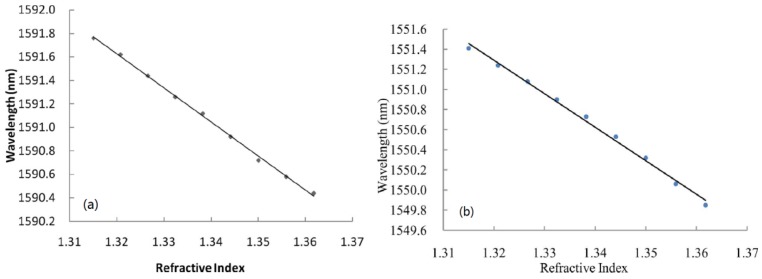
The RI sensitivity of two in-line MIs based on: (**a**) the fiber taper; (**b**) the core-offset structure, respectively.

So far, several kinds of in-line MIs have been reported based on special fibers, for instance, reference [75] fabricated an in-line MI by simply splicing a section of MMF to SMF, and reference [80] proposed an in-line MI which consists of a section of two-core fiber (TCF) and SMF. Meanwhile, the in-line MIs based on air-holes collapsing of the PCF have been demonstrated by reference [86,90,91]. Such kind of PCF-based MIs have higher RI sensitivity and low temperature sensitivity due to the special air-hole silicon structures. However, these in-line MIs require special fibers, which make it expensive to use in industry. On the contrary, the other ways to achieve the low-cost in-line MIs in SMF include LPFG [60,61], fiber taper [67], core-offset structure [70]. Although the LPFG can realize coupling and re-coupling between the core and cladding modes in the MI, the fabrication of the LPFG require precise (and often expensive) photolithographic alignment equipment and amplitude masks. Figure 13 shows two in-line MIs formed in SMF based on the fiber taper and core-offset structures, respectively. As shown in Figure 13(a), light in the core will be partially coupled into the cladding by the abrupt taper and gradually attenuated. However, since a layer of gold is coated on the fiber end facet, the core and cladding modes will be reflected by the mirror and then be re-coupled together by the same abrupt taper. In Figure 13(b), the core-offset structure has the same effects on coupling and re-coupling between the core and the cladding modes. Figure 14 shows that two in-line MIs with the same interferometer length of ∼38 mm have similar RI sensitivities of ∼290 nm/RIU (based on taper) and ∼333 nm/RIU (based on core-offset), which are comparable to that of a pair of LPFGs based MZI (∼252 nm/RIU) [59].

**Figure 15.**
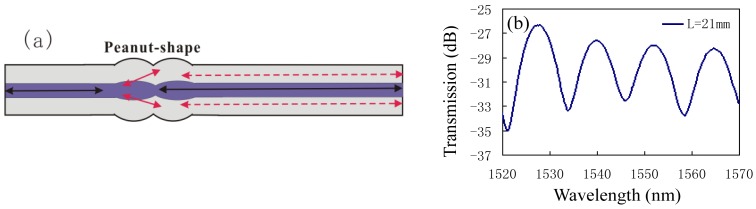
The peanut-shape based MI with *L* = 21 mm: (**a**) schematic; (**b**) reflection spectrum.

**Figure 16.**
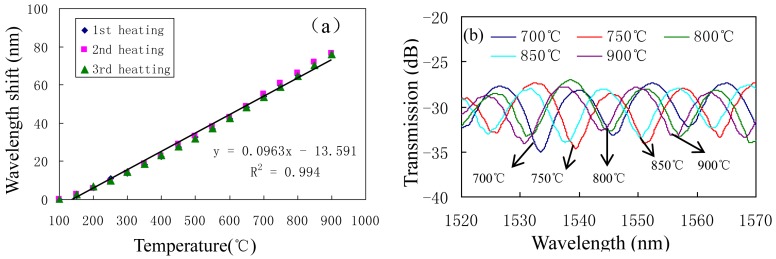
Interference fringes of the peanut-shape based MI with *L* = 22 mm at different temperatures: (**a**) temperature sensitivity; (**b**) transmission spectra.

In reference [69], Wu *et al.* have demonstrated that a novel peanut-shape fiber structure can excite high-order cladding modes and recouple the cladding modes to the core mode, thus, an in-line MI in SMF can also be formed by using only one peanut-shape structure to play the roles of splitter and combiner, as shown in Figure 15(a). Figure 15(b) shows the interference spectra of the peanut-shape based on the in-line MI with interference length of ∼21 mm, and the typical interference fringe has a visibility around 10 dB. Since the core of fiber has a higher thermo-optic coefficient than that of the cladding, such kind of MI has much high temperature sensitivity of ∼0.096 nm/°C, as shown in Figure 16(a). We can see that in the three heating cycles, the responses of the resonant wavelength to temperature are very stable, linear, and repeatable. Meanwhile, Figure 16(b) shows that there is no obvious deterioration of the spectrum in the temperature range of below 900 °C, which means that the peanut-shape based MI is an attractive high temperature sensor. Compared with other proposed in-line MI temperature sensors based on SMF/MMF [75] and LPFGs [61], the peanut-shape based MI sensor shows much higher temperature sensitivity with the simpler fabrication and the lower cost.

Overall, since the fiber optic in-line MI requires only one coupler to use as the splitter/combiner and detects the reflected signal, the measurement system of the MI will be simpler than that of MZI. The fiber taper based and core-offset based MIs have the similar RI sensitivity with LPFG, while are lower cost than it. However, they have a degraded mechanical strength due to the small waist diameter and misaligned spliced joint. The peanut-shape based MI could be used as a high temperature sensor with high sensitivity, simple fabrication, and low cost. But, it should be further improved, such as decreasing the size, coating film at the end of the fiber to reduce the insert loss.

## 4. Conclusions

In the past few decades, optical fiber sensors have been widely used in sensing applications of various physical, chemical, and even biological measurements. Among them, the in-line fiber optic interferometers have the advantages of compact structure, good stability, and easy fabrication. We reviewed the two types of typical in-line fiber optic interferometers (FPI and CCMI) formed in SMFs. Some methods for fabricating in-line fiber optic interferometers based on SMFs are described according to their operating principles, fabrication methods, and application fields. In particular, several recently reported in-line fiber optic interferometers are presented in detail to show their advantages and the great potential sensing applications. In conclusion, the in-line fiber optic interferometers based on SMFs have two obvious advantages of low cost and compact structure, which make them attractive for optical communication and sensing applications in industry.
